# Cardiac sympathetic denervation could be associated with dysphagia in Parkinson's disease

**DOI:** 10.3389/fneur.2022.1010006

**Published:** 2022-10-11

**Authors:** Jinyoung Youn, George Umemoto, Eungseok Oh, Jinse Park, Wooyoung Jang, Yoon-Sang Oh, Hee-Tae Kim, Jin Whan Cho, Shinsuke Fujioka, Yoshio Tsuboi

**Affiliations:** ^1^Department of Neurology, Samsung Medical Center, School of Medicine, Sungkyunkwan University, Seoul, South Korea; ^2^Swallowing Disorders Center, Fukuoka University Hospital, Fukuoka, Japan; ^3^Department of Neurology, Chungnam National University College of Medicine, Chungnam National University Hospital, Daejeon, South Korea; ^4^Department of Neurology, Inje University, Haeundae Paik Hospital, Busan, South Korea; ^5^Department of Neurology, Gangneung Asan Hospital, University of Ulsan College of Medicine, Gangneung, South Korea; ^6^Department of Neurology, College of Medicine, The Catholic University of Korea, Seoul, South Korea; ^7^Department of Neurology, Hanyang University College of Medicine, Seoul, South Korea; ^8^Department of Neurology, Faculty of Medicine, Fukuoka University, Fukuoka, Japan

**Keywords:** Parkinson's disease, dysphagia, ^123^I-metaiodobenzylguanidine (MIBG), non-motor symptoms, VFSS, swallowing

## Abstract

**Background:**

Dysphagia is an important non-motor symptom that is closely associated with quality of living and mortality in Parkinson's disease (PD). However, the pathophysiology of dysphagia in PD remains inconclusive. We tried to confirm whether the occurrence of dysphagia could be related to sympathetic degeneration using cardiac ^123^I-metaiodobenzylguanidine (MIBG) scintigraphy.

**Methods:**

We prospectively recruited 27 PD patients and classified them into two groups (PD with dysphagia vs. PD without dysphagia) by Swallowing Disturbance Questionnaire (SDQ) score and compared the clinical characteristics, videofluoroscopic swallowing study (VFSS) findings and parameters from cardiac MIBG scintigraphy.

**Results:**

The mean early and late H/M ratios were significantly lower in the PD with dysphagia group than those in the PD without dysphagia group (1.39 ± 0.21 vs. 1.86 ± 0.21, *p* < 0.01; 1.26 ± 0.18 vs. 1.82 ± 0.29, *p* < 0.01). In the correlation analysis, both the early and late H/M ratios were negatively correlated with the SDQ score and total VDS score (*r* = −0.65, *p* < 0.01; *r* = −0.53, *p* < 0.01; *r* = −0.65, *p* < 0.01, *r* = −0.58, *p* < 0.01).

**Conclusion:**

We confirmed that cardiac sympathetic denervation might be associated with the presence and severity of dysphagia. This finding indicates that dysphagia in PD could be associated with a nondopaminergic mechanism.

## Introduction

Dysphagia in Parkinson's disease (PD) is a frequent non-motor symptom that is closely associated with quality of living and mortality (1). Although it is known that prominent dysphagia is a relatively late symptom in disease progression, many studies have reported that mild dysphagia could be frequent in early-stage PD subjects (1–3). Kalf et al. reported that the pooled prevalence of subjectively oriented reports of dysphagia was 35%, but the prevalence of dysphagia increased to 82% when using objective methods (4). Therefore, dysphagia might be underestimated due to a lack of awareness, and it is important to identify dysphagia and its clinical predictors.

The pathophysiology of dysphagia in PD remains poorly understood. Generally, the dopaminergic pathway has an important role in the supramedullary system, but pathologic substrates in nondopaminergic brain areas that contribute to the swallowing process might also be involved in the development of dysphagia (2, 5, 6). Many studies revealed that cognitive dysfunction could be inversely associated with swallowing ability, and Suntrup et al. reported reduced cortical activation in temporal regions in PD subjects with dysphagia (7). Furthermore, Lee et al. revealed that central cholinergic dysfunction could also be associated with dysphagia in PD patients, and Mu et al. reported that peripheral motor and sensory nerves innervating pharyngeal muscles in PD patients with dysphagia showed alpha-synuclein deposition compared to those without dysphagia (5, 8).

Thus, assuming that various non-motor symptoms in PD could be clustered to some extent and that non-motor features in similar clusters might share pathophysiologic mechanisms, dysphagia in PD patients, which might have very heterogeneous and complex pathomechanisms, could be related to other non-motor symptoms, such as dementia, affective symptoms, and autonomic dysfunction (9).

^123^I-Metaiodobenzylguanidine (MIBG) is a physiological analog of guanethidine, and it is taken up and stored in sympathetic neurons in a similar way to norepinephrine and thus non-invasively allows one to assess the functional activity of postganglionic presynaptic cardiac sympathetic neurons in PD patients (10, 11). Therefore, cardiac imaging with ^123^I-MIBG is widely regarded as useful method for evaluating the integrity and activity of cardiac sympathetic innervation and the heart to mediastinum (H/M) ratio is widely used as parameter for ^123^I-MIBG uptake (12). Reduced uptake of ^123^I-MIBG in myocardial sympathetic neurons has been reported in PD and has been demonstrated to be useful for the early identification of PD and differentiation from other types of parkinsonism (13). Generally, uptake of cardiac MIBG is known for its inverse correlation with age of onset, bradykinesia, rigidity, and axial symptoms (14). Furthermore, many nondopaminergic non-motor symptoms, such as REM behavior sleep disorder, visual hallucination, dementia, and olfactory dysfunction, were reported to have a lower H/M ratio, reflecting cardiac sympathetic dysfunction, than that in PD without those non-motor symptoms (15–17). Therefore, we could hypothesize that cardiac MIBG scintigraphy in patients with dysphagia would yield lower values than those in PD patients without dysphagia if dysphagia in PD shares the pathophysiology of adrenergic and cholinergic non-motor symptoms and that, if so, cardiac MIBG scintigraphy would be one of the clinical predictors of PD-related dysphagia.

In this study, we classified enrolled PD patients according to the clinical scale, Swallowing Disturbance Questionnaire (SDQ), into PD with or without dysphagia groups and then compared the clinical characteristics, videofluoroscopic swallowing study (VFSS) findings and parameters from cardiac MIBG scintigraphy between the two groups.

## Patients and methods

### Patients and clinical assessment

All participants were enrolled from the Movement Disorder Clinic of Gangneung Asan Hospital, Samsung Medical Center, and Fukuoka University Hospital from January to August 2019. All PD patients gave written informed consent, and this study was approved by each local ethical committee.

We prospectively recruited 10 PD patients with subjective dysphagia using the Swallowing Disturbance Questionnaire (SDQ). The SDQ is a self-report questionnaire that comprises 15 items. We defined PD with dysphagia as PD patients showing SDQ scores of more than or equal to 11. Next, 17 age-matched PD patients without dysphagia were also enrolled according to the SDQ score. The exclusion criteria were (1) history of medical disorders affecting swallowing function and cardiac ^123^I-MIBG scans, including traumatic brain or spine injury, autoimmune disease, stroke, DM, congestive heart failure, and history of coronary artery disease; (2) history of medication affecting swallowing function and cardiac scintigraphy results; and (3) presence of red flag signs indicating atypical parkinsonism.

Basic demographic data, including gender, age, body mass index, disease duration and levodopa equivalent daily dose (LEDD), were assessed. The disease severity was evaluated by the “on” state Unified Parkinson's Disease Rating Scale-III (UPDRS-III), the Korean-Version of the Non-motor Symptoms Scale for PD (NMSS-K) and the Hoehn and Yahr stage. The MoCA-K and BDI scores were also evaluated in all PD patients.

### VFSS

To evaluate swallowing function in PD subjects, the VFSS was performed according to the following protocol. First, all subjects were told to keep the contrast food and/or liquid in the mouth with upright posture until the instruction to swallow was delivered. After the instruction was given, rice gruel, ground fruit, and egg custard containing the contrast medium were placed into the mouth with a spoon, and 3, 5, or 8 ml of the contrast medium was added into the subject's mouth with a syringe. Rice gruel, ground fruit, and egg custard are corresponding to the level of five based on the International Dysphagia Diet Standardization Initiative (IDDSI) Framework (18). The fluorography imaging was recorded on a videotape running at a rate of 15 frames/s with a videocassette recorder (Sonialvision, KF-7) coupled to a countertimer that placed timing information on each video field (19). The fluoroscope was activated, and the order to swallow was immediately given. All subjects underwent swallowing evaluation with 3, 5, and 8 ml of thick liquid and 3, 5, and 8 ml of thin liquid. The subject performed one swallow of both the solid food and the liquid containing the contrast medium.

The videotapes were analyzed frame-by-frame in slow motion to assess the videofluoroscopic dysphagia scale (VDS) (20). The VDS scoring was performed by an independent rater, and this rater was blinded to the subjects' group assignments.

The VDS consisted of 14 items that were divided into oral stage function (bolus formation, mastication, apraxia, premature bolus loss, and oral transit time) and pharyngeal stage function (pharyngeal triggering, vallecular and pyriform sinus residue, laryngeal elevation and epiglottic closure, pharyngeal coating, pharyngeal transit time, and aspiration) (21). We calculated the sums of the oral and pharyngeal stages, which yielded three parameters (overall oral stage score, overall pharyngeal stage score, and total VDS score).

### ^123^I-MIBG cardiac scintigraphy

Cardiac ^123^I-MIBG scans were performed 15 min (early image) and 4 h (late image) after the intravenous injection of an average of 111 MBq (3 mCi) ^123^I-MIBG using a dual-head gamma camera (ECAM, Siemens Medical Systems, Chicago, IL, USA). The cardiac and mediastinal regions of interest were observed for the semiquantification of ^123^I-MIBG uptake, and the heart-to-mediastinum (H/M) uptake ratios and washout rates (WRs) were calculated using the following formulas: early H/M ratio = mean count of the heart uptake at 15 min/mean count of the mediastinum uptake at 15 min; late H/M ratio = mean count of the heart uptake at 4 h/mean count of the mediastinum uptake at 3 h; WR = [mean counts of the heart uptake at 15 min-(mean counts of the heart uptake at 4 h × 1.1735 for decay correction)] × 100/mean counts of the heart uptake at 15 min.

### Statistical analysis

All data were analyzed with a commercial statistical software program (SPSS 12.0, Chicago, IL, USA). Comparisons of categorical and continuous variables were performed by the Mann–Whitney test and Fisher's exact test, respectively. The correlations between the H/M ratio and severity of swallowing dysfunction were explored by correlation analyses. All data are expressed as the means and standard deviations.

## Results

The demographic features of all PD subjects are shown in Table 1. There were no significant differences in age, gender, disease duration, BMI, UPDRS-III, BDI, or LEDD between the two groups. However, H&Y stage and MoCA-K score showed significant difference between two groups. The SDQ and total VDS scores were significantly higher in the PD with dysphagia group than those in the PD without dysphagia group (13.50 ± 3.82 vs. 3.41 ± 2.55, *p* < 0.01; 15.20 ± 8.22 vs. 6.00 ± 3.21, *p* < 0.01). Total NMSS-K score was significantly higher in PD with dysphagia group (38.70 ± 17.35 vs. 24.76 ± 13.82, *p* < 0.05). For each domain, cardiovascular, sleep/fatigue, attention/memory, and gastrointestinal domain revealed significant difference between two groups (Table 2).

**Table 1:** The demographic parameters of the enrolled PD subjects.

	**PD with dysphagia (*n* = 10)**	**PD without dysphagia** **(*n* = 17)**	***p*-value**
Age	71.12 ± 5.78	74.40 ± 8.53	0.16
Gender (male/female)	5/5	11/6	0.34*
Disease duration (years)	7.92 ± 5.16	8.94 ± 4.98	0.55
BMI (kg/m^2^)	23.78 ± 4.16	24.39 ± 2.38	0.56
UPDRS-III	24.17 ± 6.53	20.06 ± 4.07	0.28
Modified HandY	2.35 ± 0.24	1.97 ± 0.48	**<0.05**
LEDD	815.58 ± 224.97	715.58 ± 187.56	0.13
Beck depression scale	12.80 ± 12.63	8.71 ± 4.93	0.17
MoCA-K	23.30 ± 4.16	22.88 ± 3.41	**<0.05**
SDQ score	13.50 ± 3.82	3.41 ± 2.55	**<0.01**
Total VDS score	15.20 ± 8.22	6.00 ± 3.21	**<0.01**

These values represent the means ± the standard deviation or the number of patients, with percentages in parentheses. *Chi-squared test. PD, Parkinson's disease; BMI, Body mass index; UPDRS-III, Unified Parkinson's Disease Rating Scale Part 3; HandY, Hoehn and Yahr stage; LEDD, L-dopa equivalent daily dose; MoCA-K, Korean version of Montreal cognitive assessment; SDQ, Swallowing Disturbance Questionnaire; VDS, videofluoroscopic dysphagia scale. Bold: variables with *p*-value < 0.05.

**Table 2:** Comparison of NMSS-K score between PD with dysphagia and PD without dysphagia group.

**NMSS domain**	**PD with dysphagia (*n* = 10)**	**PD without dysphagia** **(*n* = 17)**	***p*-value**
Cardiovascular	3.46 ± 0.84	2.17 ± 1.42	**<0.05**
Sleep/Fatigue	7.10 ± 1.20	3.94 ± 2.66	**<0.05**
Mood	6.60 ± 3.41	4.41 ± 3.52	0.13
Perceptual problems	2.60 ± 1.51	1.29 ± 1.21	0.33
Attention/Memory	6.00 ± 2.11	3.18 ± 2.92	**<0.05**
Gastrointestinal	4.60 ± 1.51	3.06 ± 0.49	**<0.05**
Urinary	3.20 ±1.40	2.47 ± 0.50	0.33
Sexual function	1.50 ± 1.18	1.18 ± 1.47	0.54
Miscellaneous	3.70 ± 2.50	3.06 ± 2.11	0.48
Total NMSS	38.70 ± 17.35	24.76 ± 13.82	**<0.05**

These values represent the means ± the standard deviation. NMSS-K, Korean version of non-motor symptoms scale; PD, Parkinson's disease. Bold: Statistically significant.

The comparison of VFSS evaluation between PD with dysphagia and PD without dysphagia revealed significant differences in both the oral and pharyngeal phases (Table 3). The overall oral stage score was higher in PD patients with dysphagia than that in PD patients without dysphagia (3.75 ± 2.67 vs. 1.32 ± 0.50, *p* < 0.05). In the pharyngeal phase, the triggering of pharyngeal swallow component score was significantly higher in PD patients with dysphagia than that in PD patients without dysphagia (1.35 ± 1.17 vs. 0.00 ± 0.00, *p* < 0.05). The total VDS score was also higher in the PD with dysphagia group than that in the PD without dysphagia group (15.20 ± 8.22 vs. 6.00 ± 3.21, *p* < 0.01).

**Table 3:** Comparison of each subitem in the oral and pharyngeal stages obtained by a videofluoroscopic swallowing study (VFSS) between the PD with dysphagia and PD without dysphagia groups.

	**PD with dysphagia**	**PD without dysphagia**	***p*-value**
**Oral stage**			
Lip closure	0.80 ± 1.03	0.00 ± 0.00	ns
Bolus formation	0.90 ± 1.45	0.00 ± 0.00	ns
Mastication	0.00 ± 0.00	0.00 ± 0.00	ns
Apraxia	0.75 ± 0.79	0.00 ± 0.00	ns
Tongue to palate contact	0.00 ± 0.00	0.00 ± 0.00	ns
Premature bolus loss	1.83 ± 0.66	1.50 ± 0.00	ns
Oral transit time	0.00 ± 0.00	0.00 ± 0.00	ns
Overall oral stage	3.75 ± 2.67	1.32 ± 0.50	**<0.05**
**Pharyngeal stage**			
Triggering of pharyngeal swallowing	1.35 ± 1.17	0.00 ± 0.00	**<0.05**
Vallecular residue	2.00 ± 0.94	1.41 ± 0.94	ns
Laryngeal elevation	0.00 ± 0.00	0.00 ± 0.00	ns
Pyriform sinus residue	2.70 ± 2.32	2.91 ± 2.22	ns
Coating on the pharyngeal wall	1.80 ± 3.79	0.00 ± 0.00	ns
Pharyngeal transit time	0.00 ± 0.00	0.00 ± 0.00	ns
Aspiration	3.60 ± 5.06	0.35 ± 1.46	ns
Overall pharyngeal stage	11.45 ± 10.44	4.68 ± 2.88	ns
Total score	15.20 ± 8.22	6.00 ± 3.21	**<0.01**

Mann–Whitney test for comparison of each variable.

These values represent the means ± the standard deviation or the number of patients, with percentages in parentheses. PD, Parkinson's disease. Bold: variables with *p*-value < 0.05.

**Figure 1:**
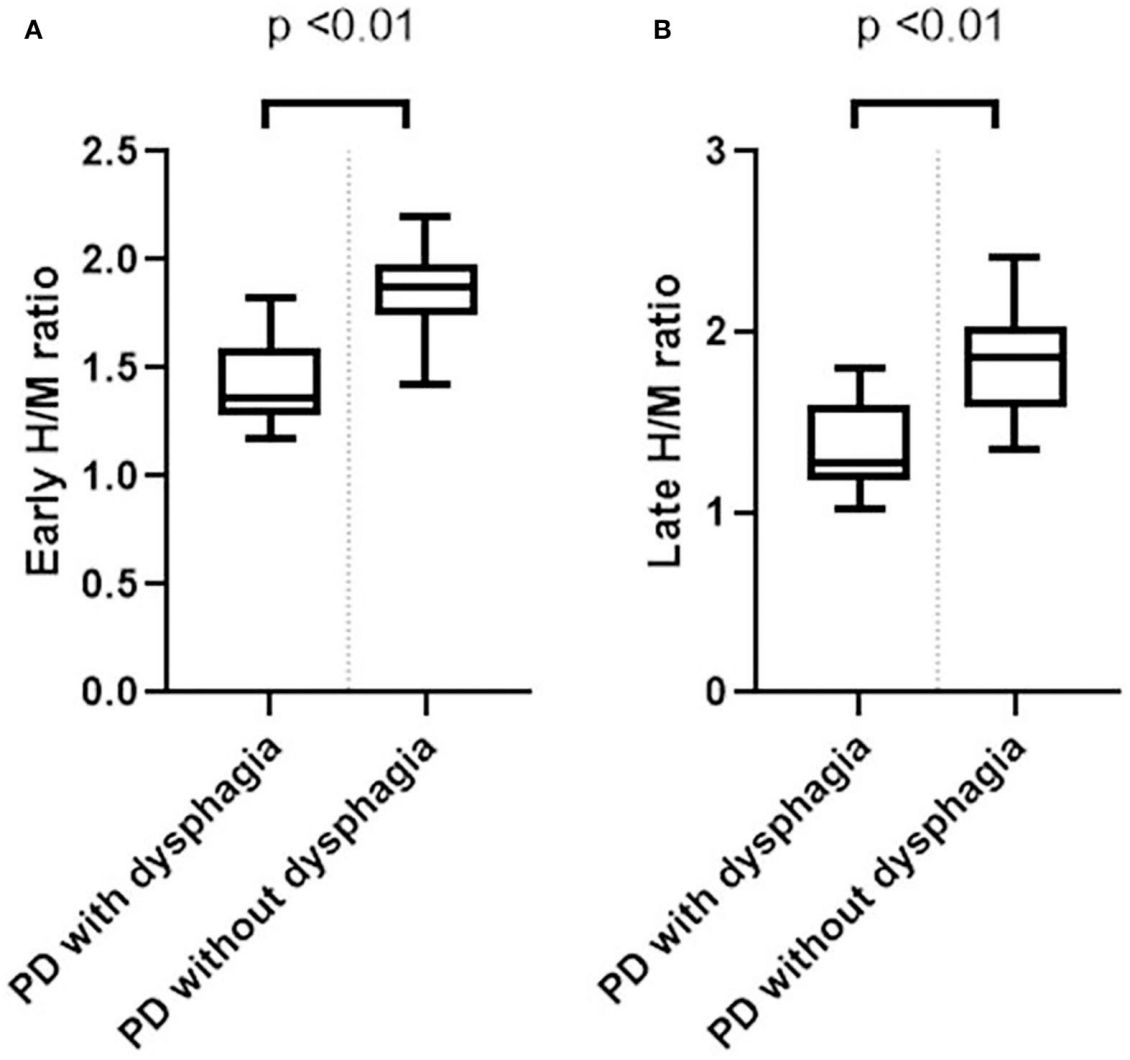
Box plots of the early **(A)** and late **(B)** H/M ratios between the PD with dysphagia and PD without dysphagia groups.

**Figure 2:**
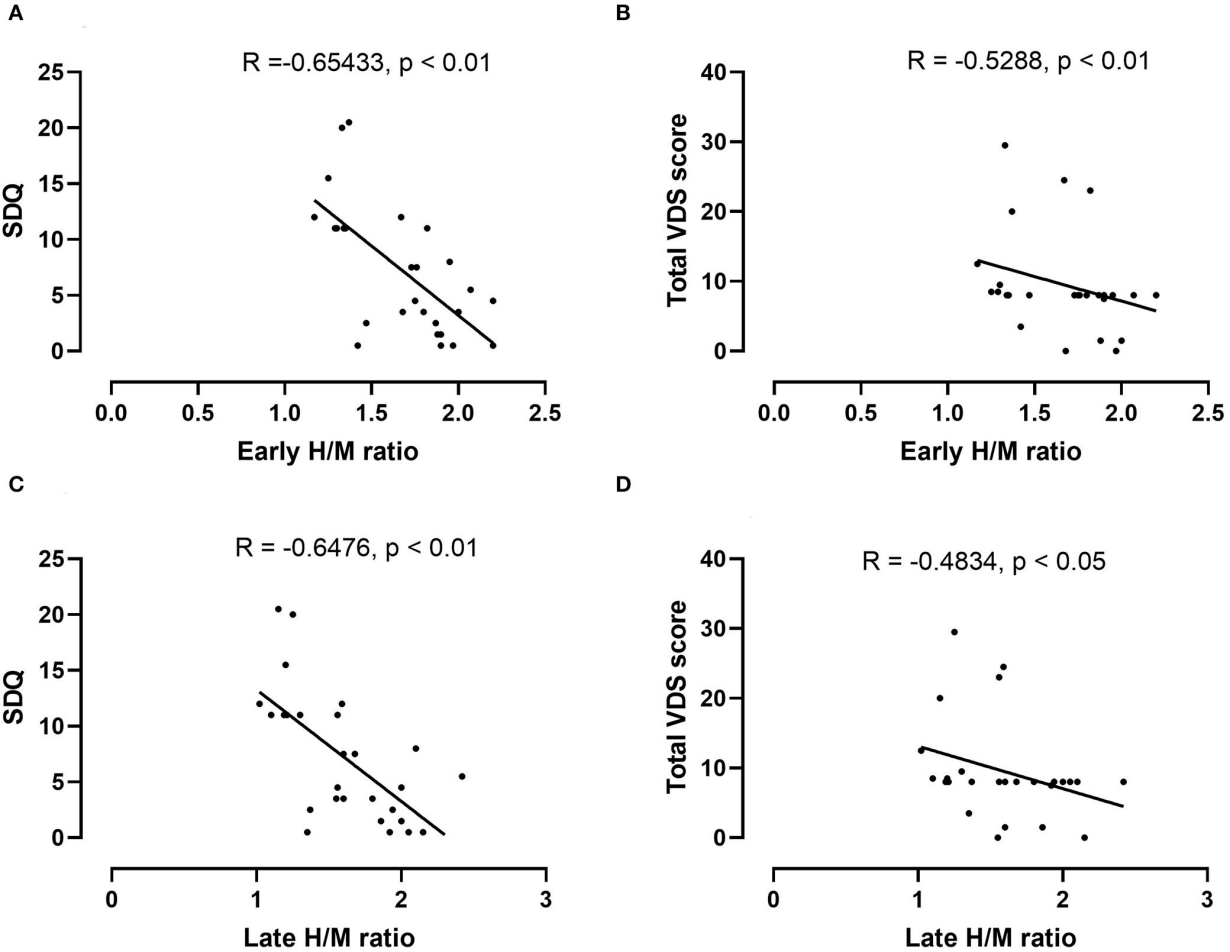
Correlation analysis between the early H/M ratio and the SDQ **(A)** and total VDS scores **(B)** in the PD dysphagia group. There was a significant negative correlation between the early H/M ratio and the SDQ (*r* = −0.65, *p* < 0.01) and total VDS scores (*r* = −0.53, *p* < 0.01). In the correlation analysis between the late H/M ratio and the SDQ **(C)** and total VDS scores **(D)**, the late H/M ratio also shows a similar negative correlation with the SDQ (*r* = 0.65, *p* < 0.01) and total VDS scores (*r* = −0.48, *p* < 0.05).

The mean early and late H/M ratios showed significant differences between the two groups (Figure 1). The mean early and late H/M ratios were significantly lower in the PD with dysphagia group (1.39 ± 0.21 vs. 1.86 ± 0.21, *p* < 0.01; 1.26 ± 0.18 vs. 1.82 ± 0.29, *p* < 0.01). The WR between the two groups did not reveal a statistically significant difference. In the correlation analysis, both the early and late H/M ratios were negatively correlated with the SDQ score and total VDS score (*r* = −0.65, *p* < 0.01; *r* = −0.53, *p* < 0.01; *r* = −0.65, *p* < 0.01, *r* = −0.58, *p* < 0.01) (Figure 2).

## Discussion

In this study, we confirmed that PD subjects with dysphagia might have more severe cardiac sympathetic denervation and that the severity of dysphagia could also be correlated with the degree of functional integrity of the cardiac sympathetic nerve. This finding indicates that the mechanism of dysphagia in PD could be associated with that of other non-motor symptoms, such as cholinergic or noradrenergic dysfunction and alpha synuclein deposition beyond the striatonigral system.

Many studies reported that non-motor symptoms could be categorized, and Kim et al. suggested that gastrointestinal symptoms in PD could be clustered with perceptual and cardiovascular symptoms (22). Furthermore, Mu et al. identified four PD subtypes according to the clustering of motor and non-motor symptoms (23). Taken together, these studies suggest that PD with prominent dysphagia might be a different pathogenetic subtype than PD without dysphagia. Therefore, MIBG uptake reflecting cardiac sympathetic dysfunction could be represented differently according to subtype. Our study also showed significant difference in various non-motor domains according to presence of dysphagia and our result is consistent with this hypothesis.

There is also substantial evidence that MIBG uptake could be different in PD subgroups with specific symptoms. Kitayama et al. reported that a reduction in MIBG cardiac uptake could be related to visual hallucination, and Nomura et al. also reported a relationship between reduced MIBG uptake and RBD (16, 17). Regarding dementia and cognitive dysfunction in PD, reduced cardiac MIBG uptake has been suggested as a predictive marker as well as a differential indicator (24). Considering that dementia, psychiatric symptoms and RBD are closely related to central cholinergic dysfunction, these reports indicate that cardiac sympathetic dysfunction in PD could be closely coupled with simultaneous, more widespread involvement of multiple neurotransmitter systems rather than greater neuronal loss in only the peripheral sympathetic nervous system. Furthermore, we previously reported that dysphagia in PD could be associated with cholinergic dysfunction (5). Therefore, it is plausible that dysphagia in PD is associated with reduced cardiac MIBG uptake and that cardiac MIBG scintigraphy could be an indicator of PD-related dysphagia.

Although the pathophysiology of dysphagia in PD remains unclear, swallowing problems in PD might be evident in all phases of swallowing, and both dopaminergic and nondopaminergic mechanisms seem to be involved in the development of dysphagia in PD. Regarding the role of the dopaminergic pathway in swallowing, it has been revealed that basal ganglia structures are activated during swallowing in normal healthy subjects using functional MRI (25). Furthermore, dopaminergic medication and deep brain stimulation reveal significant improvement of swallowing function in PD (3, 26). In this study, all PD subjects were taking dopaminergic medication. Therefore, the characteristics of dysphagia and its significant correlation with MIBG uptake in this study might be specific to PD-related dysphagia with a nondopaminergic mechanism.

Pathologic substrate accumulation in the relevant areas could be suggested as the nondopaminergic mechanism of dysphagia in PD. Gastrointestinal dysfunction has been proposed as one of the prodromal symptoms in PD prior to the onset of motor symptoms, and some studies found evidence of alpha-synuclein deposition in colonic tissue before the development of motor symptoms (27). Similarly, Braak staging reported the propagation of alpha synuclein pathology affecting the dorsal nucleus of IX and V and the locus coeruleus in the premotor phase of PD (28). Therefore, dysphagia in PD could be derived from Lewy body pathology, which is involved in relevant areas for swallowing impairment, such as medullary swallowing centers. Sakibahara et al. suggested a close association between reduced cardiac MIBG uptake and premotor symptoms of PD, such as autonomic failure, cognitive dysfunction, depression and sleep disorder (24). These prodromal non-motor symptoms are also explained by aberrant Lewy body pathology involvement prior to substantia nigra pathology. Therefore, our finding of the association between reduced MIBG uptake and dysphagia in PD also supports the plausibility of the mechanism by which the pathological substrate of PD in the brain stem or cortical area relevant to swallowing function leads to the development of swallowing problems in PD patients. Furthermore, Sung et al. described impairment of the esophagus even in the early stage of PD, and Mu et al. reported alpha synuclein involvement in peripheral motor and sensory nerves innervating pharyngeal muscle in PD patients with dysphagia (8, 29).

We are aware that there were many limitations in this study. First, the small number of patients is the main limitation of our study. Second, we did not recruit a healthy control group or atypical parkinsonism group for comparing with PD. Swallowing physiology could change as aging process and sarcopenia and reduced connective tissue elasticity can lead to various negative impacts on swallowing mechanism (30). Chen et al., recently reported swallowing function in sarcopenic elderly subjects was significantly impaired before evidence clinical manifestation (31). Therefore, we cannot confirm whether our result was specific to the PD group or not. Third, we enrolled PD dysphagia patients using a subjective scale. There are several reports of discrepancies between subjective dysphagia and objective evaluation. Therefore, it is possible that enrollment of the subjective dysphagia group did not completely reflect a PD dysphagia group. Finally, it is potentially possible that PD without dysphagia group could include prodromal multiple system atrophy (MSA) patients, although we tried to exclude atypical parkinsonism patients by exclusion criteria including various clinical red flag signs (unequivocal cerebellar abnormalities, downward vertical supranuclear palsy, no response to high-dose levodopa, cortical sensory symptoms or ideomotor apraxia/aphagia, absence of common non-motor symptoms) (32). Many studies revealed that cardiac MIBG scintigraphy could differentiate PD from MSA with high sensitivity and sensitivity (10, 33). However, Druschky et al. reported MIBG uptake in MSA patients was also decreased compared to healthy controls (34). Considering that recent criteria for possible prodromal MSA required presence of at least one of RBD, Neurogenic OH, or Urogenital failure and presence of at least one of subtle parkinsonism or cerebellar sign, it could be overlapped with early stage PD with prominent specific non-motor symptoms (35). Unexplained anosmia could be exclusion criterion for prodromal MSA, but we did not perform olfactory function test in this study. Therefore, further study including subjects satisfying possible prodromal MSA criteria would be required for resolving this limitation.

In conclusion, although the mechanism of dysphagia in PD is very complex, cardiac sympathetic denervation might be associated with the presence and severity of dysphagia, especially *via* a nondopaminergic mechanism. Cardiac MIBG scintigraphy could be a useful method for identifying subclinical dysphagia in PD patients.

Longitudinal studies might be warranted for cardiac MIBG scintigraphy as a clinical predictor of dysphagia in PD.

## Data availability statement

The raw data supporting the conclusions of this article will be made available by the authors, without undue reservation.

## Ethics statement

The study was approved by the Ethical Committee of Gangneung Asan Hospital, Samsung Medical Center, and Fukuoka University Hospital. The patients/participants provided their written informed consent to participate in this study.

## Author contributions

WJ and SF proposed the research idea, performed the data analysis and interpretation, and wrote the manuscript. EO, JP, Y-SO, H-TK, JC, and YT provided the clinical suggestions. JY and GU conceptualized and designed the research, wrote the manuscript, and prepared the manuscript for submission. All authors contributed to the article in a meaningful manner.

## Funding

This research was financially supported by the Gangneung Asan Hospital Biomedical Research Center promotion fund. The funder was not involved in the study design, collection, analysis, interpretation of data, the writing of this article or the decision to submit it for publication.

## Conflict of interest

The authors declare that the research was conducted in the absence of any commercial or financial relationships that could be construed as a potential conflict of interest.

## Publisher's note

All claims expressed in this article are solely those of the authors and do not necessarily represent those of their affiliated organizations, or those of the publisher, the editors and the reviewers. Any product that may be evaluated in this article, or claim that may be made by its manufacturer, is not guaranteed or endorsed by the publisher.
